# Enhancing doctor-patient relationships in community health care institutions: the Patient Oriented Four Habits Model (POFHM) trial—a stepped wedge cluster randomized trial protocol

**DOI:** 10.1186/s12888-023-04948-w

**Published:** 2023-06-29

**Authors:** Yunying Zhu, Sisi Li, Ruotong Zhang, Lei Bao, Jin Zhang, Xiaohua Xiao, Dongdong Jiang, Wenxiao Chen, Chenying Hu, Changli Zou, Jingna Zhang, Yong Zhu, Jianqiu Wang, Jinchun Liang, Qian Yang

**Affiliations:** 1 School of Public Health, and Department of Geriatrics of the Fourth Affiliated Hospital, Zhejiang University School of Medicine, Zhejiang University, Hangzhou, Zhejiang Province 310058 China; 2 Community Health Service Center in Jiangcun Street, Hangzhou, 310050 Zhejiang Province China; 3 Community Health Service Center in Sandun Town, Hangzhou, 310030 Zhejiang Province China; 4 Community Health Service Center in Liuxia Street, Hangzhou, Zhejiang Province 310050 China; 5 Xu Zhen Town Center Health Center, Wuhu, 241306 Anhui Province China; 6 Community Health Service Center in Jishan Town, Wuhu, 241307 Anhui Province China; 7 Nanling County Traditional Chinese Medicine Hospital, Wuhu, 241307 Anhui Province China

**Keywords:** Doctor-patient communication, Patient literacy, Sense of control, Stepped wedge cluster randomized trial, Four habits model, Theoretical domain framework

## Abstract

**Background:**

The poor relationship between doctors and patients is a long-standing, global problem. However, current interventions tend to focus on the training of physicians, while patient-targeted interventions still need to be improved. Considering that patients play a significant role in outpatient consultations, we developed a protocol to assess the effectiveness of the Patient Oriented Four Habits Model (POFHM) in improving doctor-patient relationships.

**Methods:**

A cross-sectional incomplete stepped-wedge cluster randomized trial design will be conducted in 8 primary healthcare institutions (PHCs). Following phase I of “usual care” as control measures for each PHC, either a patient- or doctor-only intervention will be implemented in phase II. In phase III, both patients and doctors will be involved in the intervention. This study will be conducted simultaneously in Nanling County and West Lake District. The primary outcomes will be evaluated after patients complete their visit: (1) patient literacy, (2) sense of control and (3) quality of doctor-patient communication. Finally, a mixed-effects model and subgroup analysis will be used to evaluate the effectiveness of the interventions.

**Discussion:**

Fostering good consultation habits for the patient is a potentially effective strategy to improve the quality of doctor-patient communication. This study evaluates the implementation process and develops a rigorous quality control manual using a theoretical domain framework under the collective culture of China. The results of this trial will provide substantial evidence of the effectiveness of patient-oriented interventions. The POFHM can benefit the PHCs and provide a reference for countries and regions where medical resources are scarce and collectivist cultures dominate.

**Trial registration:**

AsPredicted #107,282 on Sep 18, 2022; https://aspredicted.org/QST_MHW

**Supplementary Information:**

The online version contains supplementary material available at 10.1186/s12888-023-04948-w.

## Background

Conflicts between patients and doctors are still a prevalent problem worldwide [1, 2]. The incidence of workplace violence committed by patients and visitors against healthcare workers was prevalent globally, as high as 61.9% [1]. In China, 55.73% of healthcare workers and 33.70% of patients believe their relationship is tense [3].

Good communication is important for fixing difficult relationships between doctors and patients. Because both doctors and patients cite "good doctor-patient communication (DPC)" as the top factor of improvement in the doctor-patient relationship (DPR) [4, 5]. Besides, high-quality communication between patients and doctors has been proven to facilitate treatment adherence and self-management [6], improve health outcomes [7, 8] for patients and relieve burnout for doctors [7, 9]. Therefore, various intervention strategies to improve DPC have been proposed.

Various models and theories serve as a guide for research and practice. The American scholar Keller first proposed the E4 model of DPC (Engagement, Empathy, Education, Enlistment) and criticized the treatment process that centres on the disease [10]. Frankel then developed the Four Habits model (FHM) in 1996 [11], which involves investing in the beginning, eliciting the patient’s perspectives, demonstrating empathy, and investing in the end. In response to the call for an improved evaluation of communication skills, Makoul reported the SEGUE framework, a research-based checklist of DPC tasks [12]. SEGUE is an acronym for five areas (Set the stage, elicit information, give information, Understand the patient's perspective, and end the encounter), covering the entire medical interview [12]. From a theoretical perspective, shared decision-making (SDM) has recently become the focus of research. SDM emphasizes patient-centeredness, where patients and physicians are partners with equal power [13]. In addition, targeted theoretical tools have been developed for different scenarios, for example, prompt question lists for cancer patients [14] and patient activation for chronic patients [15].

Training for doctors and medical students has been the primary focus of DPC interventions and has developed a relatively mature training system from concept to practice [16]. Researchers now recognize the binary nature of the DPR and the critical role of patients [17]. However, most studies still tend to promote patient involvement in medical decision-making by educating physicians rather than patients [18], which is due in significant part to doctors’ dominant position in the treatment [19]. A scoping review reveals that nearly 50% of identified SDM interventions only targeted physicians [20]. Whereas the reliance on physician authority increases the risk of physicians acting as scapegoats. Previous studies have reported that patients who lack a sense of control tend to blame physicians for their health problems [21]. Furthermore, it is challenging to balance dominance and share decision-making with patients in communication if only doctors are skilled [22]. As the saying goes, "it takes two to tango", the expectation that effective communication could result from interventions aimed solely at either the doctor or the patient was unrealistic. One study found that SDM led to doubts about the doctor's competence for some patients, reflecting patient literacy heterogeneity [23]. In short, the process of DPC is dynamic in which doctors and patients interact. However, interventions to educate patients have been limited to date [24]. Therefore, the priority of our research protocol is to improve patient literacy, enhance the sense of control, and promote DPC through educating patients, followed by minor intervention with doctors.

The intervention design in this study was developed from the four-habit model (FHM). A longitudinal study has shown that FHM-based communication skills are closely and positively correlated with patient satisfaction scores [25]. Recently, based on a similar theoretical framework, Ming Tai-Seale’s team developed a multidimensional intervention called OpenComm to promote more open communication between patients and doctors [26]. Nevertheless, OpenComm focuses on communication content and ignores the importance of paying attention to emotions [26]. The competence of actively noticing emotional changes can be exercised through brief mindfulness intervention [27]. Based on the FHM, combined with mindfulness theory and the concept of patient-centeredness, we developed a complete framework including four good habits of patients and doctors, called Patient Oriented Four Habit Model (POFHM). The first habit (invest in the beginning) corresponds to patient role activation, an essential precursor to SDM, requiring knowledge, skills, and confidence in self-health management [28]. The second habit (focus on the greatest concern) corresponds to the question priority order, which was helpful in facilitating DPC [14, 29]. The third habit (focus on emotions) corresponds to emotional attention. The evidence suggests that over 90% of the studies reported a significant positive effect of brief mindfulness-based interventions on at least one health-related outcome [30]. The fourth habit (invest in the end) is a restatement, which helps the patient remember and follow the doctor's advice.

In addition, a consolidated framework to follow implementation science is crucial to bridging the gap between research evidence and practice [31]. Therefore, to ensure that the interventions work best in practice, the theoretical domain framework (TDF) was introduced to assess potential facilitators and barriers to intervention implementation [32]. Moreover, considering the characteristics of collectivist culture and Confucian culture, the role of the patient is often more than one person but a family [33, 34]. Therefore, the scope of the term "patient" in this study encompasses not only individuals receiving medical treatment but also their family members and friends.

In all, this study aims to evaluate the effectiveness of the POFHM intervention in primary health care institutions. POFHM is a promising intervention that integrates the four-habit theory, mindfulness theory, and patient-centered concept, providing education on four habits for mainly patients and then doctors during medical visits. The program ultimately expected to enhance the doctor-patient relationship in China, based on primary healthcare institutions. Initially, this study will evaluate the effectiveness of POFHM intervention, followed by conducting a manipulation check and process evaluation, in accordance with the implementation science framework [35]. Therefore, the results of POFHM can serve as a valuable reference for regions with limited medical resources and collectivist cultures.

In order to achieve full coverage of beneficial interventions and assess the impact of interventions, we will employ a stepped wedge cluster randomized trial (SW-CRT) [36]. The stepped wedge design enables the effectiveness of intervention programs to be tested systematically in a controlled manner. Firstly, clusters in a stepped wedge design effectively transition from the control group to the intervention group. At each phase, outcomes are evaluated among study participants in all clusters, ensuring that each cluster contributes data in both the control and intervention conditions [36]. Secondly, the stepped wedge design is advantageous as it permits researchers to progressively introduce and assess the various components of an intervention, while continuously optimizing the intervention [36]. Examples of stepped wedge investigations include the efficacy of 3 cancer pain guideline implementation strategies [37] and effects of a preschool-based sleep health literacy program [38]. The POFHM intervention is brief, and patients are unlikely to have repeat medical visit in the short term. Therefore, this study uses a cross-sectional and incomplete SW-CRT [39, 40], with a specific study design presented below.

## Methods

### Participants

#### Study sites

There were two primary reasons for selecting the implementation site. Firstly, we acknowledged that the economic level and the distinction between urban and rural areas may impact the efficacy of POFHM intervention. Secondly, we aim to verify that the intervention is effective for the majority of Chinese. To ensure that our sample is both representative and universal, we plan to recruit four PHCs in each of our chosen regions, Nanling County and West Lake District in China, which display significant differences. Please refer to Table 1 for an overview of the key components of the stepped wedge design.

**Table 1:** Key characteristics of the POFHM trial

Trial characteristics	Definition
Cluster (unit of randomization)	Hospitals or Primary health care facilities (a total of 8)
Number of sequences (steps)	4 (2 hospitals per sequence)
Duration of trial	1 month
Number of measurement periods	7 (length of each period is 3 days)
Individuals	Patients (greater than or equal to 16 years old) visit a doctor at any time during the study
Timing of start of exposure	Patients are exposed to the process when they wait to be seen
Duration of exposure	Patients are exposed for a short period during their visit to the doctor
Measurement	Repeated measurements are from mostly different patients in each period; a tiny proportion of individuals may have repeat visits to the same hospital

The scope of the patient concept in this study extends beyond just the patients themselves and includes their family members and friends

#### Eligibility criteria for hospitals (clusters) and doctors

Hospitals and doctors are eligible for inclusion in the study if: (1) The primary healthcare facilities see at least 100 patients daily. (See sample size calculation); (2) We will recruit doctors from enrolled PHCs willing to participate in the project. To facilitate the arrangement of the investigation schedule, doctors whose daily patient volume is less than 30 or whose clinic hours are irregular will be excluded.

#### Eligibility criteria for patients

In order to cover as many patients as possible, we will enroll patients above the age of 16 and family members accompanying them during their visit. During the study period, these patients will visit the enrolled doctors at least once.

#### Intervention development and description

The interventions adopted in the study are effective in tertiary care hospitals. Implementing scientific assessments identified obstacles and promoting factors, integrating the intervention with routine procedures reduces implementation difficulty, although doctors still experienced an increase in workload, as detailed in the pilot study [41] On this basis, the interventions were adapted and piloted to the characteristics of primary healthcare facilities. First, we conducted semi-structured interviews with doctors in PHCs, revealing that primary care facilities' patient population is predominantly elderly with chronic illnesses and children with common illnesses. Doctors believed that whether patients trust them has the most significant impact on DPC. The lack of patients' health literacy would impair the effect of treatment and undermine doctor-patient trust. Therefore, we adapted the interventions based on these findings to promote DPC in primary care settings.

We then identified feasible implementation methods. Considering physicians' workload and primary healthcare facilities' primary patients, we determined to recruit volunteers to deliver the intervention to patients. During the waiting period, volunteers will engage with patients in the waiting area, provide pre-visit education on the POFHM, and administer a questionnaire after their visit.

### Interventions and control

In China, the relationship between doctors and patients is mainly authoritative [19], and patients lack initiative in the treatment process [42]. This relationship can lead to the consequences of patients not paying attention to self-health management and putting all the blame on doctors [43], which will reduce medical effectiveness and damage doctor-patient trust over time [44, 45]. Therefore, the first good habit of training patients is to activate the patient role, making them realize their essential position and role during treatment.

Even if patients are aware of their role, not all can clearly express their needs [19]. Therefore, it is necessary to train a second good habit of patients to organize their thoughts and identify their most important concerns for their current visit.

The emotional states of doctors and patients inevitably affect communication in the treatment process [46]. Negative emotions can magnify malice, damage the DPR, intensify doctor-patient conflicts, and cause medical disputes [46]. Many studies focus on training healthcare professionals to recognize patients' emotions and provide them with empathetic and supportive care [47, 48]. However, little attention has been paid to patients' role in DPC. Awareness of one's emotions is a prerequisite for controlling them [49]. Therefore, the third good habit of training patients is to be aware of their own and others' emotions. Finally, actively confirming important information with the doctor and remembering medical advice is the fourth good habit that needs to be trained in patients.

Based on the scientific evaluation results of the preliminary trial implementation, it was found that minimal intervention with doctors helps them to better play the supporting role in the research plan [41]. Therefore, this study design will further simplify the FHM interventions for doctors. The four good habits for doctors and patients are detailed in Table 2.

**Table 2:** Patient-oriented four habits model (POFHM) intervention description

Habits	Patient (major)	Doctor (minor)
Habit 1: invest in the beginning	Understand the importance of the patient's role	Create rapport quickly
Habit 2: focus on the greatest concern	Sort through the issues of greatest concern	Know what the patient is most concerned about
Habit 3: focus on emotions	Pay attention to emotions	Accept and channel the patient's emotions
Habit 4: invest in the end	Actively summarize information	Summary and feedback

#### Intervention 1: FHM only for patients

The POFHM intervention program for patients consisted of four good habits during visits (see Table 2). The intervention plan was not implemented by making booklets (although we did this in the previous plan) but by recruiting volunteers to ask patients questions to implement the intervention. On the one hand, the potential subjects' visual impairment and limited educational level were considered. On the other hand, hearing a question that requires an answer can better promote patients' thinking, which is the critical point of the intervention design. Below are the detailed implementation points for the four habits.

##### Habit 1: invest in the beginning

The first step of the intervention plan is to activate the patients. We designed four short judgment questions as the first part of the intervention measures. Through questions from volunteers, we encourage patients to reflect on their responsibilities in the treatment process. Volunteers provide straightforward explanations, such as "Seeing a doctor is not only the responsibility of the doctor; patients should also be responsible for their health!", "Doctors cannot cure all diseases like many chronic diseases cannot be cured; doctors can only help you to alleviate or delay the disease " to prompt or deepen the patient's awareness of their role as a patient. The key points of the intervention are (1) patients thinking after hearing the questions and (2) patients recognizing the importance of the " patient's role" after hearing the answers. We do not care about the correctness of the answers.

##### Habit 2: focus on the greatest concern

The second step of the intervention is to help patients sort out their thoughts and identify their most important concerns for this visit. We designed 3–4 short questions as the second part of the intervention. Volunteers delivered the intervention by asking patients questions. The critical point of the intervention is that "patients think after hearing the questions", and we do not care about the specific content of the answers.

##### Habit 3: focus on emotions

In this study, we used the core mindfulness theory to design the intervention's third step. Two simple single-choice questions, such as "Do you have the following feelings now?", "Emotions can be as contagious as a virus"; was used to awaken the patient's awareness of self and others' emotions to make the DPC more harmonious.

##### Habit 4: invest in the end

Based on the initial interview results, we provided the patients with some options for reference, such as retelling, drawing circles, and underlining. While volunteers assign the task to patients, they also emphasize that we will be checking on completing the task.

#### Intervention 2: FHM only for doctors

The researchers will explain the contents of the FHM intervention to the doctors individually and provide complete prompting guidance in writing to intervene with the medical staff. Volunteers are responsible for maintaining order in the waiting room while the patient is waiting and conducting a questionnaire survey after the patient completes the visit. The specific components are shown in Table 2.

#### Intervention 3 (POFHM): FHM both for doctors and patients

The primary focus of the complete POFHM is to cultivate patient visit habits and encourage doctors to provide positive feedback for patients. As we emphasized in the previous section, the construction of the DPR is the result of the joint efforts of both sides. Therefore, the researchers hypothesized that complete POFHM, targeting both doctors and patients, would achieve the best effects.

#### Control group

The essence of the incomplete stepped wedge design is that all primary healthcare facilities entered the study, starting with the control group and then entering different interventions in a randomly assigned sequence for the rest of the study period. During the control period (phase I), the doctors will complete routine consultations according to their habits. Volunteers will be responsible for maintaining order in the waiting room while the patient is waiting and conducting a questionnaire survey after the patient has completed the visit. The written guidance for doctors and the interactive question-and-answer training for patients will only be implemented when they enter the intervention period (phase II and III).

### Primary outcomes

This patient-oriented, physician-supported intervention incorporates the sense of control theory in its design. The intervention focuses on improving patient literacy, with the ultimate goal of effectively improving the quality of DPC.

#### Sense of control

Sense of control will be measured using the 6-item internal health locus of control form A which was developed by Wallston in 1978 [50]. The internal health locus of control form A has been found to have acceptable levels of validity and reliability [51].

#### Patient literacy

Patient literacy will be measured using a patient literacy scale developed by Jiang (2022) [52]. However, the knowledge dimension of the scale was removed because it is not the focus of our attention. Moreover, the items "I will make a doctor's appointment online before my visit" and "When I see the reports of doctor-patient conflicts, I would like to verify" are removed because they do not meet the conditions of the field in a preliminary investigation.

#### Doctor-patient communication

Doctor-patient communication will be measured using 10-item consultation and relational empathy (CARE), which was developed by Mercer et al. in 2004 [53]. The CARE has been widely used in the field of patient-doctor relationships and has demonstrated acceptable levels of validity and reliability [54].

### Sample size

According to our previous work, an absolute increase of 0.13 or more in DPC scores for patients is meaningful. Our sample size of eight clusters (1272 patients) achieves 80% power to detect a 0.13 absolute difference using a two-sided test at the 5% significance level [55, 56]. Our calculation assumes an intra-cluster correlation coefficient of 0.034 [57], an average of 53 patient encounters per site in each 3-day interval. While our study is a cluster RCT, given the cross-sectional nature of a stepped-wedge design and brief intervention period, the effect of individual autocorrelation coefficients (IAC) and cluster autocorrelation coefficients (CAC) on power is negligible, therefore we did not adjust for IAC and CAC [39, 58]. In conclusion, we aim to include 1590 patients to account for a degree of missing data.

### Recruitment

We will recruit a convenience sample of practices from Nanling County and West Lake District. We will then arrange an in-person meeting with leaders and family physicians from interested sites to introduce our study and obtain a written agreement from PHCs and doctors. Patients will be recruited when they arrive at these PHCs.

### Randomization and blinding

#### Randomization

**Fig. 1:**
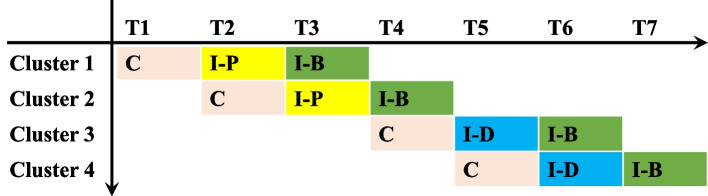
Stepped-wedge study design. C, control; I-P, FHM intervention only for patients; I-D, FHM intervention only for doctors; I-B, POFHM intervention both for doctors and patients

Each participating PHC stratified by region will be randomized to one of the predefined timelines (as depicted in Fig. 1) using a single sequence of random assignments. The research assistant will communicate intervention starting times with the participating PHCs.

#### Blinding

Given the features of SW-CRT, blinding doctors and researchers is impossible. Patients will be re-recruited at each phase, and they are blind to the allocation scheme. Analyses will be performed by a researcher blinded to the PHCs allocation scheme.

### Study procedures

During the preparation phase, standard operating procedures (SOPs) for each phase have been developed according to characteristics of PHCs, including investigation process and norms, questionnaire survey instructions and training manuals. A pre-survey will then verify the validity and accuracy of the questionnaire and the survey plan. In addition, we will recruit local people experienced in the investigation as volunteers and then conduct unified training for SOPs. During the implementation phase, volunteers will deliver the intervention to patients. At the same time, the research assistant will do it to doctors, and the research assistant will be on-site throughout to ensure the intervention implementation process is standardized and uniform.

### Data collection procedures

All patients will be informed of the study information orally and in writing before the investigation or intervention. Their data will be collected only after providing written informed consent. All information about patients and doctors will be collected by questionnaire. The data collection norm is described in the SOPs. Paper questionnaires will be double entered through EpiData 3.1 version to ensure the accuracy of the information. Electronic questionnaires will be exported through the online "Questionnaire Star" platform (https://www.wjx.cn).

To better understand any potential factors that may explain the trial results, a qualitative evaluation based on TDF will be conducted after the trial is completed to assess family doctors' and patients' perceptions and experiences about the trial.

### Statistical analysis

We will use cluster-specific methods because randomization will be performed at the PHC level. For all primary outcomes, an intention-to-treat analysis will be performed. To evaluate the effects of the primary outcomes of the three interventions, generalized linear mixed models will be used, specifying the PHC effect as random and the time effect as fixed [59, 60]. Multiple imputations using chained equations will be used in case of missing data. Subgroup analyses will be conducted to evaluate the interventions' effect in different regions. All analyses will be carried out using Stata 17.0 software. A 2-tailed error rate of 5% will be used to test the hypothesis.

## Discussion

This paper has outlined the study design of the patient-oriented four habits model intervention and data collection. As far as we know, this study is the first to use an SW-CRT trial design to evaluate the effectiveness of a patient-oriented intervention designed to improve DPC. The conflict between patients and doctors is still an urgent problem, especially in China [61]. The intervention based on the patient-oriented four habits model is one of the effective measures to promote DPR [62]. It is despite the relative vulnerability of the patient during the medical visit [19]. However, access to more psychological resources and knowledge skills can increase the patient's sense of control during the visit [63], precisely what POFHM focuses on.

This trial has several important strengths. First, we developed universal interactive interventions based on previous research to provide practical patient training. In addition, to ensure that the interventions developed in this study work best in practice, the TDF was introduced to assess potential facilitators and barriers to intervention implementation. Second, the stepped-wedge design benefited all participating PHCs. Simultaneously, we utilized the stepped-wedge design to assess the difference in the effectiveness of a relationship intervention that involved either the doctor or the patient alone, versus involving both of them. Third, Subgroup analyses will provide a new perspective on understanding the heterogeneity of intervention effects in regions.

A potential limitation of this trial is that blindness is not allowed, as every PHC experienced a shift from a control group to an intervention group. However, it will be almost impossible for patients to be recruited repeatedly. Furthermore, we will reduce the personal impact of investigators and information bias by formulating a standard operating procedure. Another limitation of our study is the difficulty in sustaining the recruitment of volunteers to implement the intervention in routine situations. This emphasizes the need for further development of an interactive voice electronic app.

In conclusion, this study will provide helpful information on the effectiveness and universality of the patient-oriented, doctor-supported intervention.

## Supplementary Information

**Additional file 1.**

**Additional file 2.**

## Data Availability

Not applicable.

## Abbreviations

SDM: Shared decision-making
FHM: Four habits model
POFHM: Patient Oriented Four Habits Model
PHCs: Primary healthcare institutions
DPC: Doctor-patient communication
DPR: Doctor-patient relationship
TDF: Theoretical domain framework
SOPs: Standard Operating Procedures
SW-CRT: Stepped Wedge Cluster Randomized Controlled Trial
